# Social Participation for Health and Health Care Workers’ Self-Management: Lessons from Socialist Yugoslavia for Contemporary Health Systems—A Systematic Review

**DOI:** 10.3390/healthcare14182933

**Published:** 2026-09-09

**Authors:** Predrag Duric, Smiljana Rajcevic, Jelena Djekic Malbasa, Dunja Prokic, Ljiljana Milosevic

**Affiliations:** 1 Faculty of Environmental Protection, Educons University, 21208 Sremska Kamenica, Serbia; dunja.prokic@educons.edu.rs (D.P.); ljiljana.curcic@educons.edu.rs (L.M.); 2 Faculty of Pharmacy Belgrade, Educons University, 11000 Belgrade, Serbia; 3 Faculty of Medicine, University of Novi Sad, 21137 Novi Sad, Serbia; smiljana.rajcevic@mf.uns.ac.rs (S.R.); jelena.djekic-malbasa@mf.uns.ac.rs (J.D.M.); 4 Institute of Public Health of Vojvodina, 21102 Novi Sad, Serbia; 5 Institute of Pulmonary Diseases of Vojvodina, 21204 Sremska Kamenica, Serbia

**Keywords:** health systems, health policy, health system reform, participatory governance, self-management, decentralisation, universal health coverage, social solidarity, Yugoslavia

## Abstract

Background/Objectives: Contemporary health systems continue to face persistent challenges in financing, equity, and governance, particularly under conditions of limited resources, workforce shortages, and demographic pressures. While the World Health Organization calls for strengthening social participation to achieve universal health coverage, evidence suggests that such participation often remains largely symbolic. Existing global examples of social participation in health are limited either by their short duration of implementation or by their limited scope. In contrast, the experience of former Yugoslavia (1945–1991) represents a distinctive case. In the early post-war period, health reforms were directed towards the development of a decentralised, self-managed health system that emphasised universal coverage, participatory governance, social solidarity, and local autonomy. The trajectory of these reforms was further shaped by global economic and political pressures, including neoliberal trends, international debt, and the oil crises, which constrained resources and exposed systemic vulnerabilities. The so-called Yugoslav Experiment may offer an alternative to existing market-oriented and, at times, inefficient health systems. This review aims to synthesise evidence on the governance, financing, organisation, and outcomes of Yugoslavia’s self-managed health system and to evaluate its relevance to contemporary debates on health system resilience, decentralisation, participatory governance, equity, and sustainability. It examines power relations, stakeholder interests, and resource allocation, and assesses the lessons that the Yugoslav experience offers for contemporary debates on participatory governance and the sustainability of healthcare systems, while identifying areas of convergence, debate, and gaps in the existing scholarship. Methods: We conducted a systematic narrative review of peer-reviewed articles, book chapters, conference papers, and commentaries published in English or Bosnian–Croatian–Montenegrin–Serbian that covered the period 1945–1991. Scopus, PubMed, and EBSCOhost (including CINAHL Plus with full text, Medline and PsychInfo) were searched on 19 January 2026. Studies were included if they addressed health system reform, self-management, governance, financing, or institutional continuity. Contextual studies of selected services—primary care, mental health, reproductive health, and vaccination—were included only if they illuminated reform trajectories. Data synthesis focused on reform phases, institutional transformations, financing arrangements, governance mechanisms, and power relations, with particular attention to continuity, path dependence, and critical junctures. Given the heterogeneous and predominantly historical, analytical, and policy-oriented nature of the literature, we did not apply conventional study-design-specific risk-of-bias tools. Instead, we used an overarching framework to classify and contextualise the included evidence according to five dimensions: source type, nature of contribution, evidentiary role, temporal context, and health-system domain. The protocol was not registered. The study was financed by the Provincial Secretariat for Higher Education and Scientific Research, Autonomous Province of Vojvodina, Republic of Serbia. Results: The database searches yielded 265 records, of which 193 were selected for title and abstract screening after removing 72 duplicates. At this stage, 133 publications were excluded because they did not meet the eligibility criteria and 60 publications were selected for full-text review. Application of the predefined inclusion and exclusion criteria resulted in 22 eligible publications. An additional 18 studies were identified through citation chaining and targeted Google Scholar searches. Twenty-two publications were coded as primary studies, directly examining health system reform, governance, or self-management, while 18 were classified as contextual studies addressing specialty areas. The analysis identified key reforms that followed the social participation, i.e., ‘self-management’, approach, which expanded primary care, preventive services, and local participation. It revealed institutional changes, as well as the role of power and health financing in health reforms aimed at strengthening social participation. Nonetheless, challenges—including bureaucratic and managerial elitism, unequal professional authority, resource scarcity, and tensions between market-oriented policies and social solidarity—were amplified by global economic forces and neoliberal trends, ultimately contributing to the system’s collapse by the late 1980s. This review was limited by its search strategy, the exclusion of grey literature, the potential omission or inaccessibility of relevant studies, and its focus on selected aspects of health reform, namely post-war recovery and self-management. Conclusions: The Yugoslav experience illustrates both the promise and the fragility of socially oriented, participatory health systems, demonstrating how international political alignments and global economic pressures can profoundly shape domestic health reform trajectories. Lessons from this case remain relevant to contemporary efforts to strengthen social participation for universal health coverage through designing sustainable, resilient, equitable, and socially accountable health systems under complex global conditions.

## 1. Introduction

The last three and a half decades have represented a period of profound health reforms at the global level. The end of the Cold War and the collapse of the Eastern European bloc triggered extensive reform processes across Central and Eastern Europe in the early 1990s, but also globally [1]. Rapid globalisation, together with international initiatives aimed at combating HIV, tuberculosis, malaria, and other major diseases, generated new demands for adaptation within national health systems. At the same time, comprehensive reform programmes promoted by international financial institutions, particularly the World Bank and the International Monetary Fund, had a significant influence on health policy design and implementation in many countries [2,3].

Ten years ago, the World Health Organization acknowledged that functional health systems delivering high-quality services to the population are a key priority for governments, and that achieving this requires permanent, well-structured, and dynamic processes, underpinned by genuine consensus between the demand for and supply of services, as well as among governments, service providers, and the population, through robust, evidence-informed policy dialogue [4]. Social participation is defined as “empowering people, communities, and civil society through inclusive participation in decision-making processes that affect health, across the policy cycle and at all levels of the system” [5]. The COVID-19 pandemic further highlighted the fundamental importance of social participation in fostering trust in governments and public institutions, demonstrating that regular and institutionalised dialogue, in which people feel heard, are empowered to express their views, and are able to influence decisions, strengthens trust, supports effective COVID-19 responses, and advances the right to health [6].

Three main areas of scholarly interest in participatory governance and healthcare have been identified: the relationship between community participation and the improvement and tailoring of healthcare services; the involvement of patients and their families in therapeutic practices; and comparative analyses of case studies focusing on participatory governance in healthcare policymaking [7]. Although valuable studies have examined experiences of participatory governance in healthcare in countries including Brazil [8], Thailand [9], South Korea [10], and elsewhere [5,6], their findings are often limited in two respects: either the implementation period was relatively short, or social participation was confined to only certain aspects of healthcare. Furthermore, almost all of these studies focus on the participation of patients and citizens, while only rarely addressing the participation of healthcare workers in decision-making and the management of health institutions and health systems.

Although the available studies mainly discuss social participation for health from the early 1990s onwards, this phenomenon has a much longer history. In former Yugoslavia, it existed for nearly forty years before the country dissolved in 1991, in a specific form known as (workers’) self-management. While it was initially introduced in industrial complexes, it spread to health institutions in the early 1950s. Over the following decades, it evolved continuously and represented a form of social experiment, sometimes called ‘Yugoslav experiment’ [11].

In its mature phase during the 1970s, it included a complex framework of social participation and participatory governance, participatory decision-making processes, and transformed health institutions to ensure the direct participation of each worker in decision-making, as well as the involvement of the local community. The long duration of this specific type of social participation in health, together with the many avant-garde approaches that were introduced, allowed it to be monitored, measured, and evaluated. It attracted widespread interest among Western and local scholars concerned with participatory democracy and worker involvement [11,12,13,14,15,16]. Yugoslavia’s openness towards the West and its sustained economic growth encouraged researchers to study not only its healthcare system but also broader aspects of its society. Self-management was frequently cited as a key factor underlying the country’s perceived successes [15,16].

This systematic narrative review aims to synthesise evidence on the governance, financing, organisation, and outcomes of Yugoslavia’s self-managed health system and to evaluate its relevance to contemporary debates on health system resilience, decentralisation, participatory governance, equity, and sustainability. It examines power relations, stakeholder interests, and resource allocation and assesses the lessons that the Yugoslav experience offers for contemporary debates on participatory governance and the sustainability of healthcare systems, while identifying areas of convergence, debate, and gaps in the existing scholarship.

## 2. Materials and Methods

### 2.1. Setting

Yugoslavia was formed in 1918 from territories previously under Austro-Hungarian, Ottoman, and, until the end of the 18th century, Venetian rule for centuries, with Serbia and Montenegro having been internationally recognised as independent in 1878. Prolonged foreign domination left the new state economically underdeveloped, predominantly agrarian, and characterised by high mortality and illiteracy rates. The interwar period was marked by economic, political, and ethnic tensions.

The Second World War resulted in an estimated 1.7 million deaths, around 250,000 orphans, and destruction of nearly one-third of all buildings [17]. Yugoslav partisans, led by the Communist Party, assumed power in 1944–1945. Following the 1948 break with the Soviet Union, Yugoslavia pursued an independent socialist path, later establishing a system of workers’ self-management. Economic crises in the 1970s contributed to growing political instability and rising nationalism, culminating in the country’s dissolution in 1991, with conflicts continuing into the early 2000s.

### 2.2. Methodological Approach

The decision to adopt a systematic narrative review was deliberate and informed by the nature of the research question and the characteristics of the available literature. Our aim was to systematically identify, evaluate, and synthesise the existing evidence while allowing for an interpretive narrative synthesis, given the heterogeneity of the included studies in terms of study designs, populations, and the concepts and outcomes examined. This heterogeneity limited the suitability of a conventional systematic review with quantitative synthesis.

A scoping review was not considered appropriate because the purpose of this study extended beyond identifying and describing the range and characteristics of the existing literature. Instead, our aim was to synthesise and interpret the findings in relation to our specific research question. Similarly, a historical review was not considered appropriate because tracing the development of the topic over time was not the primary objective. Therefore, a systematic narrative approach provided the most appropriate framework for maintaining a systematic and transparent search and study-selection process, while accommodating the heterogeneous nature of the evidence and enabling an integrated narrative synthesis.

### 2.3. Search Strategy

A systematic narrative review was conducted in line with PRISMA 2020 guidelines [18] (Appendix D, Table A2). Scopus, PubMed, and EBSCOhost (including CINAHL Plus with full text, Medline and PsychInfo) were searched on 19 January 2026 using combinations of terms related to Yugoslavia, health, and reform:

“Yugoslavia” AND “health” AND (“reform” OR “crisis” OR “recovery” OR “socialism” OR “self-management”)

The search strategy was piloted prior to conducting the final searches. The strategy was assessed to determine whether it retrieved meaningful and relevant results and was also tested against a set of known relevant studies to confirm that these studies were identified. The pilot confirmed that the search strategy was appropriate, and therefore no modifications were considered necessary before conducting the final searches.

No date restrictions were applied; searches included all records up to 19 January 2026. Results were exported to Rayyan for records management.

Additional sources were identified through screening reference lists of included studies and the first ten pages of Google Scholar results to capture literature not indexed in the primary databases. Search terms and inclusion criteria were designed to capture both studies explicitly addressing health system reform and those providing contextual insights into governance, financing, professional roles, or sector-specific services.

### 2.4. Eligibility Criteria

Included publications were peer-reviewed journal articles, book chapters, conference papers, editorials, commentaries, viewpoints, and letters. Abstracts had to be available in English or Bosnian–Croatian–Montenegrin–Serbian. Studies were eligible if they explicitly discussed health system reform or provided relevant contextual information on healthcare organisation, governance, financing, or professional roles, covering fully or partially the period 1945–1991.

Excluded publications included those with unavailable full texts or in ineligible languages, studies focusing exclusively on periods outside 1945–1991 without meaningful overlap, or studies not providing substantive discussion of health systems, healthcare organisation, or institutional context. Non-peer-reviewed sources, such as dissertations, theses, news articles, interviews, as well as protocols and book reviews, were also excluded.

### 2.5. Screening and Data Extraction

All search results were imported into Rayyan, and duplicates were removed. Titles and abstracts were independently screened by two researchers, with disagreements resolved through discussion or consultation with a third reviewer. Full texts were obtained for studies meeting inclusion criteria at the title/abstract stage. Two researchers independently reviewed the full texts to ensure coverage of reform phases, self-management, governance, financing, and institutional context. Any discrepancies between the reviewers were discussed to reach a consensus. When consensus could not be achieved, a third author was consulted to facilitate resolution of the disagreement.

Data were extracted into Excel, capturing bibliographic information, temporal context, system component, source type, evidentiary role (classified as primary or contextual), nature of contribution (empirical, historical, etc.), health-system domain, institutional structures, financing arrangements, governance mechanisms, key actors and their interactions, and indicators of continuity, path dependence, or critical junctures.

### 2.6. Evidence Classification and Appraisal

Given the heterogeneous and predominantly historical, analytical, and policy-oriented nature of the literature, we did not apply conventional study-design-specific risk-of-bias tools. Instead, we used an overarching framework to classify and contextualise the included evidence according to five dimensions: source type, nature of the contribution, evidentiary role, temporal context, and health-system domain.

Studies were classified according to their evidentiary role as either primary or contextual. Primary studies were those that directly addressed health system reform, self-management, or governance under socialism. Contextual studies were eligible if they either provided in-depth analysis of specific services or specialties—including mental health, reproductive health, vaccines, or primary care—relevant to understanding reform trajectories, institutional continuity, or legacies of self-management, or provided background information on health system reform between 1945 and 1991.

The temporal context of each publication was also considered. Foundational studies covering pre-1945 developments were included when they informed postwar reform design, ideology, or institutional structures. Studies covering the post-1991 period were included when they provided insights into the legacy of self-management, institutional continuity, or adaptation of governance, financing, and service delivery arrangements.

The source type (e.g., journal article, book chapter, or conference paper) and nature of contribution (e.g., historical, review, empirical, analytical, or other) were recorded to distinguish the different forms and evidentiary bases of the literature. The health-system domain addressed by each publication was also recorded. These dimensions were used to contextualise the contribution of each source and to avoid treating heterogeneous forms of evidence as equivalent during the narrative synthesis.

### 2.7. Data Synthesis and Analysis

Data synthesis focused on mapping reform phases and critical junctures, examining institutional changes in organisation, financing, governance, and service delivery, and analysing power relations, interests, and conflicts among key actors. Primary studies formed the basis of the analysis of self-management, institutional reforms, and governance, while contextual studies complemented this analysis by highlighting sectoral, regional, and political influences.

Although the protocol was not prospectively registered, an internal protocol was developed and adhered to throughout the study.

## 3. Results

### 3.1. Overview of Included Publications

**Figure 1:**
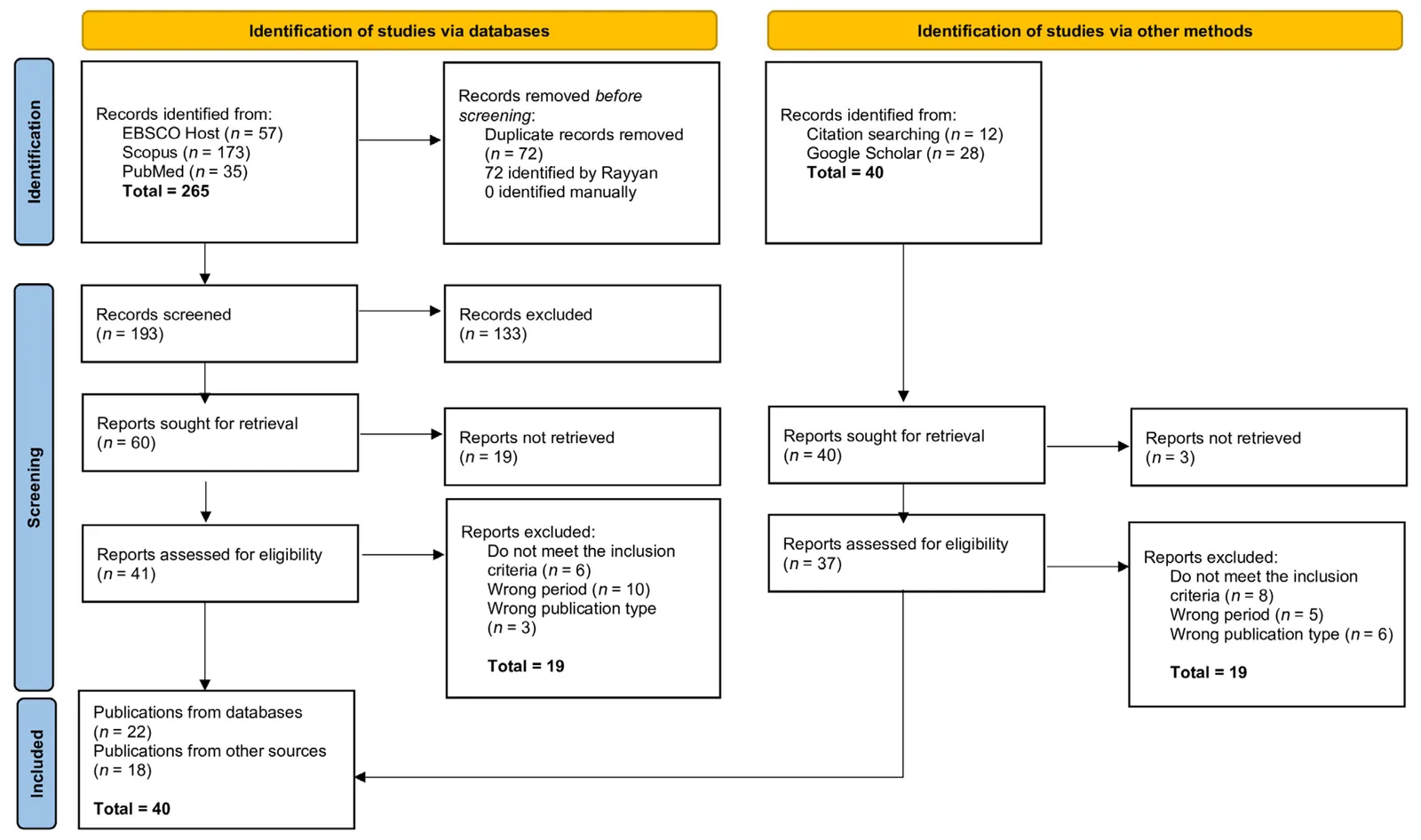
PRISMA Flow Diagram.

The database searches yielded 265 records, of which 193 were selected for screening titles and abstracts after removing 72 duplicates. At this stage, 133 publications were excluded because they did not meet the eligibility criteria and 60 publications were selected for full-text review. Applying predefined inclusion and exclusion criteria resulted in 22 eligible publications. An additional 18 studies were identified through citation chaining and targeted Google Scholar searches. In total, 40 publications were included in the final analysis (Figure 1; Appendix A; Appendix B).

The temporal distribution of publications shows that relevant literature has become increasingly available over time. More than half (52.5%, *n* = 21) were published since 2000, while 42.5% (*n* = 17) appeared between 1970 and 1999. Two publications date from the early 1950s.

### 3.2. Geographic Distribution

Based on the corresponding or first author’s affiliation, the majority of publications originated from the United States (27.5%, *n* = 11), the United Kingdom (20%, *n* = 8), and Yugoslavia (12.5%, *n* = 5) or its successor states (Serbia, *n* = 4; Croatia, *n* = 1; Slovenia, *n* = 1). The remaining publications came from Australia, Canada, Denmark, Germany, New Zealand, Spain, and the Netherlands.

### 3.3. Publication Characteristics

Most publications were journal articles (85%, *n* = 34), with the remainder being book chapters (15%, *n* = 6; Appendix C, Table A1). Considering the nature of the contribution, 24 publications were historical (including eight combined articles—e.g., analytical or empirical with a historical overview), eight empirical, whereas other publications were theoretical articles, analytical articles, viewpoints or reports. Historical studies analysed reform trajectories and institutional development using secondary sources. Empirical studies drew on primary data—such as surveys, interviews, or secondary data —administrative records—often focusing on service organisation or governance.

Regarding the evidentiary role, 22 publications were coded as primary, directly examining health system reform, governance, or self-management, while 18 were contextual, addressing specialty areas (e.g., psychiatry, reproductive health, or vaccines) or focused primarily on developments before 1945 or after 1991.

By tracing constitutional, legislative, and institutional developments across the postwar period and situating them within the broader political trajectory of the socialist state, we identified distinct reform phases corresponding to critical political junctures. Drawing on extracted data from the included studies, we mapped health sector transformations, highlighting structural innovations—such as self-management councils and communal insurance associations—and the socio-political dynamics that shaped their implementation (Table 1).

### 3.4. Wartime Period (1941–1945)

A formative wartime period (1941–1945) saw partisan medical units operating not only in liberated territories but also alongside combat forces, providing basic services and laying the foundation for postwar reform [21]. These units organised rudimentary clinics and mobile care, establishing professional networks that later underpinned postwar healthcare institutions. Despite severe resource scarcity and infrastructure destruction, partisan medicine emphasised collective responsibility, community engagement, and preventive practices. These experiences shaped postwar expectations regarding state responsibility, workforce mobilisation, and emergency health provision, influencing the institutional logic of the socialist health system [22,23].

### 3.5. Centralised Administrative Management (1945–1952: Administrative Socialism)

The first postwar phase was dominated by centralised, Soviet-inspired administration, with financing reliant on state budgets and health workers employed directly by the state [24,25,26,27,28]. Early reformers drew heavily on the interwar work of Andrija Štampar, whose vision of rural health, prevention, and social medicine guided post-1945 strategies [27,28]. Physicians, often assigned to rural or underserved areas, prioritised training and institution-building despite harsh living and working conditions [26,29,30]. Widespread outbreaks of infectious disease and profound psychological distress highlighted the scale of postwar health needs [31].

Following the 1948 Tito–Stalin split, Yugoslavia gradually moved away from Soviet-style command planning towards a distinct Yugoslav model combining socialism with decentralisation and worker participation [16,32]. While the state retained strong control, this period established the institutional and ideological groundwork for the decentralisation reforms of the 1950s and 1960s, particularly in healthcare financing and governance [16,33].

### 3.6. Early Self-Management (1953–1960: Administrative Market Socialism)

From as early as 1949, and more widely from 1953 onwards, self-management principles were introduced, initially in enterprises and progressively extended to the health sector [12,13,16,22]. Federal health planning evolved into a hybrid system, combining central guidance with increasing republic- and local-level authority, as annual and five-year plans devolved operational responsibilities to communes and institutions [13,32]. Social insurance began covering patient costs, and primary health centres (domovi zdravlja) were established to expand access and strengthen preventive services [34,35]. These centres provided universal coverage, addressed priority health problems, and cared for vulnerable groups through multidisciplinary teams embedded in local communities [36].

Workers’ councils became primary mechanisms for employee influence, holding authority to select managers from approved lists, set internal pay scales, determine hiring procedures, and allocate surpluses between wages and investment. While management guided most decisions, workers played a significant role in welfare, employment, and compensation matters [32]. Decentralised responsibility allowed communities and local governments to influence service provision, expanding the role of primary care physicians and preventive work [37]. Participatory bodies—such as workers’ councils, tenant councils, and assemblies of social service users and providers—broadened citizen and worker engagement in decision-making [38]. Challenges persisted, including limited expertise in health promotion, low professional status of primary care staff, weak citizen participation, and the continued dominance of curative, hospital-based medicine.

### 3.7. Formalised Self-Management (1961–1973: Market Socialism)

The main principles of the 1968 Law on Health Care and Health Service introduced elements of primary healthcare nearly a decade before the Alma Ata Conference [39,40]. During the 1960s and early 1970s, self-management principles were formalised within healthcare institutions, with expanded autonomy and financing increasingly reliant on workers’ and farmers’ insurance funds rather than state budgets [16,34,38]. Centrally planned state budgets were gradually replaced by “social plans,” giving enterprises greater autonomy over income, resource allocation, and market participation. While this encouraged efficiency, it also generated unemployment, emigration, and tensions between management and workers [32].

Health and other service institutions adopted a form of social self-management, with governance shared between employees and external representatives, including consumers, while budgets and staffing remained subject to oversight by councils and general assemblies [16,38].

Commune-level oversight and bicameral assemblies further institutionalised fiscal and administrative decentralisation. Efficiency gains were reported in some areas, yet tensions persisted between professional authority and worker councils, particularly regarding investment priorities and service organisation. Preventive care, mental health initiatives, and reproductive health programmes expanded during this period [24,41,42].

### 3.8. Constitutional Reforms and SMIC/BOAL Era (1974–1988: Contractual Socialism)

Following the 1974 Constitution and the Law on Associated Labour, Basic Organisations of Associated Labour (BOALs) and Health Self-Management Interest Communities (SMICs) became central governance units in the health sector [14,16]. BOALs enabled employees to participate formally in staffing, budgets, and service delivery decisions, while SMICs coordinated financing and planning across multiple institutions at the regional level. These reforms aimed to strengthen self-management and reduce reliance on market mechanisms, but they also increased institutional complexity. Economic stagnation, rising social inequalities, and growing nationalist tensions further strained coordination and equity [43,44].

Self-management during this period empowered local actors but also produced fragmentation and bureaucratic overload, particularly during fiscal crises [16]. Decentralised bargaining weakened coherent planning and reduced the capacity of social services to respond to macroeconomic shocks. The mid-1980s also saw institutional innovation and organisational experimentation, with management innovations introduced alongside worker-council governance [45].

### 3.9. Collapse Period (1989–1991)

The final phase corresponded with the disintegration of the Yugoslav federation. Political collapse, economic crises, and rising nationalism disrupted health system governance, financing, and service delivery. Worker councils, SMICs, and BOALs lost cohesion, budgets fell into deficit, and inter-republic coordination deteriorated, culminating in the near-total breakdown of previously established self-management mechanisms. The period exposed the vulnerability of decentralised institutions to political and economic shocks [11,23].

### 3.10. Forms of Institutional Change in Healthcare

Between 1945 and the 1980s, Yugoslav healthcare underwent major institutional changes. Initially, policymakers prioritised establishing a universal, centralised system of care [23,29]. Early legislation, such as the 1945 Law on Implementation of Social Insurance, centralised financing through the Central Institute for Social Insurance and initially prioritised industrial workers before extending coverage to the general population, reflecting socialist commitments to universal coverage [27,32].

The government emphasised preventive medicine and public health, targeting disease outbreaks such as typhus, malaria, dysentery, diphtheria, and tuberculosis, while promoting hygiene and health education to transform the largely rural society [21,27,33,36,38].

The 1950s and 1960s saw the establishment of community-oriented primary care centres (Dom zdravlja), staffed by multidisciplinary teams including general practitioners, paediatricians, gynaecologists, and nurses, with clearly defined preventive and consultative functions [31,33,46]. While these centres formed the foundation of primary care, preventive services remained underdeveloped, and primary care physicians often faced low prestige and poor remuneration relative to specialists [37]. Reproductive and family health services evolved progressively, culminating in the 1974 constitutional guarantee of reproductive choice [22].

A major institutional change was the introduction of workers’ self-management alongside wider economic reforms [8,11,23]. Hospitals and health centres were theoretically granted autonomy from the state and managed through worker councils and community representatives, responsible for budgeting, staffing, and planning local services [47]. Employees participated in assemblies approving budgets and selecting directors, reflecting the ideal of democratic workplace participation [8,23]. However, self-management frequently generated bureaucracy, inefficiency, and fragmentation, as local facilities pursued their own priorities, while technical managers and state representatives increasingly dominated decision-making [25,44].

Despite these challenges, Yugoslavia’s health system represents a unique attempt to combine central planning, socialised medicine, and localised self-management, combining universal coverage with worker and community involvement. Reforms extended to preventive medicine, mental health, reproductive rights, and primary care, showing how socialist goals and practical constraints shaped institutional change, including the perceived broader role of GPs in the community [12,23,31,41,48,49,50]. Coordination, resource allocation, and professional hierarchies remained challenges.

### 3.11. Health Financing

Healthcare was primarily financed through social insurance contributions, supplemented by state transfers, business donations, and user fees [28,34,35,42]. From the 1960s, decentralisation and self-management increased local influence over budgeting, service provision, and insurance coverage [12,51]. Contributions were collected at district or communal levels, and providers negotiated budgets with local social insurance bodies [40,42]. Small business owners and self-employed professionals contributed voluntarily, while specialised enterprise clinics created a parallel system bypassing community health centres, contributing to weaker general practice and preventive care [34].

The formal establishment of SMICs in health financing occurred after the 1974 Constitution, institutionalising local self-management and granting responsibility for administering insurance funds, negotiating services, and overseeing local healthcare delivery [28,35]. Decentralisation had mixed effects: regional life expectancy disparities declined, but inequalities in infant mortality increased [52]. Cost-sharing measures introduced in 1978 had limited impact on overall spending and tended to exacerbate inequities [44]. Solidarity mechanisms supported poorer regions and agricultural populations, gradually extending basic and hospital care to farmers by the 1980s [28,35]. As authority shifted between federal, republican, and local institutions, financing bodies gained or lost autonomy accordingly [12,16,38,51].

### 3.12. Power Relations

In the immediate postwar period, power was concentrated in the federal state, with the Central Institute for Social Insurance controlling financing and resources, leaving little autonomy to republics, municipalities, or local health institutions [24,25,33]. Health planning followed centralised Five-Year Plans, with limited decision-making power for communes and workers [13,37].

From 1953 through the 1960s, authority gradually shifted to republic and local levels. Social management bodies were created in health facilities and Social Insurance Institutes, though powers remained limited, and federal oversight continued [16]. Representation was uneven, with male experts overrepresented and workers and women underrepresented, limiting democratisation [16]. Federal authorities intervened when expenditures were deemed excessive, showing continued federal influence [16,42].

Between 1961 and 1974, the self-management system expanded, with communal insurance associations becoming principal financiers of health services. Federal authorities retained capacity for ad hoc intervention, while trade unions, local political elites, and enterprise representatives increased participation, though experts and elites maintained significant power [16]. Republican bureaucracies expanded authority amid Communist Party divisions, while municipal assemblies and communal organisations established control over local resources [12,16].

After the 1974 Constitution, decentralisation was institutionalised through SMICs and BOALs. SMICs oversaw municipal health financing and were governed by councils representing providers and consumers, while BOALs introduced worker self-management within enterprises and social services, allowing autonomous contract negotiation [14,40,44]. Consumer participation was strengthened through health parliaments, in which workers and citizens influenced budgeting, capital projects, and operational decisions [40].

### 3.13. Convergence, Debate, and Gaps

There is broad agreement that Yugoslavia achieved important public health gains under socialist self-management, despite structural and political constraints. Population health improved markedly, driven by reductions in infant and child mortality and infectious diseases, improvements in sanitation, nutrition, and maternal/childcare services [42]. Expansion of infrastructure—hospital beds, medical schools, and insurance coverage—also increased substantially [44].

Scholars largely agree that the Yugoslav model diverged from the Soviet Semashko system, combining decentralised community responsibility with universal coverage, affecting service availability and community engagement. The model represents a unique hybrid: centralised planning with decentralised self-management, market-based incentives with social insurance, and top-down authority with participatory governance [25,42,44].

Debates persist regarding the effectiveness of self-management. While formal structures allowed worker and citizen participation, professional elites and state regulators often dominated strategic decision-making, limiting true democratisation [14,16]. Opinions diverge on efficiency: some argue decentralisation improved responsiveness, while others highlight fragmentation and regional disparities [34,44,52].

Gaps include underexplored roles of gender and social inequality in care access and council participation, uneven development of preventive versus curative services, and limited analysis linking institutional reforms to long-term health outcomes, particularly during political and economic crises.

## 4. Discussion

Our systematic review presents the main aspects of health reform in Yugoslavia, which was guided by the principles of a specific form of social participation in healthcare—self-management. It represents an avant-garde and unique example of direct democracy in healthcare. During the mature phase of self-management, complex structures of social participation were developed, and healthcare institutions were transformed in an effort to ensure that every employee was included in the decision-making process. While the review identifies the main advantages of this particular approach to social participation, it also highlights its weaknesses. More specifically, it examines institutional change, health financing, the role of power, and the debates surrounding the effectiveness and limitations of self-management.

As stated by Chuong and O’Doherty, unlike consumerist approaches, democratic approaches explicitly address issues of power, emphasising the redistribution of power and a commitment to both individual and collective empowerment [53]. However, established forms of participation have often been largely symbolic and consultative, constrained by their tendency to operate at the micro level—through limited spatial reach and a focus on policy niches—as well as by their failure to adequately address asymmetrical power relations within and beyond participatory forums [7]. Consequently, most measures used to assess social participation involving citizens and policymakers focus primarily on the existence of participation rather than on its quality [54]. Our review found the opposite pattern: the empowerment of healthcare workers and other employees reached such an extent that it weakened healthcare institutions, while social participation at the local and subnational levels almost completely eroded national control over the healthcare system. Although the intention was to ensure that employees assumed full control over their respective units and that local communities took responsibility for healthcare governance within their communities, this often resulted in a lack of solidarity with more vulnerable groups and those with different healthcare needs.

In the immediate post-war period, Yugoslavia faced several challenges that remain relevant for many low-resource health systems today. Emerging from the devastation of the Second World War—during which 11% of Yugoslav citizens lost their lives—and soon confronted with political and economic isolation following the split with the Soviet Union, the country had to reconstruct a severely damaged health system with limited institutional and material resources [17]. This reconstruction occurred under conditions of severe material scarcity, a very limited number of qualified health professionals—often with outdated training—shortages of facilities, equipment, and medicines, and a population that, prior to the war, had been among the poorest in Europe [26,29]. Illiteracy rates exceeded 50%, most of the population lived in rural areas, and subsistence agriculture predominated [42].

However, the foundations of the post-war Yugoslav health system were not created ex nihilo. They drew heavily on interwar reforms led by Andrija Štampar, who promoted decentralised health administration, the establishment of a dense network of health institutions, and a strong focus on rural populations, health education and prevention [28,34]. Although politically sidelined in the 1930s, Štampar returned after the war to play a central role in Yugoslav health policy and in shaping the emerging World Health Organization. His vision—later described by Albrecht as “peasant internationalism”—deeply influenced the normative orientation of Yugoslav health reforms [27].

This approach materialised institutionally through the development of *domovi zdravlja* (primary health centres), community-based primary care facilities distributed across all territorial units and supported by satellite outpatient stations in smaller settlements, decades before the Declaration of Alma-Ata (acknowledged in 2018 by the Declaration of Astana) [55]. Over time, these centres integrated general practice with maternal and child health, occupational health, basic diagnostics, emergency care, dentistry, pharmacy, and selected specialist services [36,38]. In line with the ideological prioritisation of industrial workers, parallel systems of workers’ health care were also established within large enterprises or linked to health centres [25]. During the early post-war period, when infectious diseases, epidemics, and high infant and child mortality dominated the epidemiological landscape, health authorities emphasised prevention, hygiene, and health education [31]. Health was conceptualised holistically—as physical, mental, and social well-being—reflecting the belief that human beings were not merely biological units, but social and moral actors embedded in collective life [49].

In addition, the initial success of self-government in Yugoslavia was built upon widespread social enthusiasm rooted in the anti-fascist partisan resistance during the Second World War [26]. For many citizens, the end of the war represented both national liberation and the possibility of constructing a different social order, forged through mass mobilisation involving multiple Yugoslav peoples and nationalities, explicitly grounded in ideals of social justice and solidarity [31]. Within this movement, a wartime health service developed that was first tested under combat conditions and later expanded in liberated territories. Despite extreme material constraints, it provided care for the wounded and organised civilian health services, including epidemic control and health education [40,56]. These wartime experiences shaped post-war expectations regarding state responsibility for health, collective action, and the social role of medicine. These lessons may be relevant to countries currently experiencing the large-scale destruction of their healthcare systems (e.g., Ukraine and Palestine) or the collapse of both healthcare systems and wider society (e.g., Sudan).

The fact that Yugoslavia was largely liberated by its own population, rather than through external military imposition, is crucial to understanding its post-war trajectory [42]. This historical specificity, together with the 1948 conflict with the Soviet Union, created conditions for a sharp political reorientation. While the short-lived post-war centralisation modelled on Soviet administrative socialism achieved some success in emergency reconstruction, it also generated widespread dissatisfaction among civilians and former fighters [22]. The experience of importing rigid ideological models and “ready-made” solutions likely contributed to Yugoslavia’s decision to pursue an alternative path. This alternative trajectory later became known as self-management socialism. It emphasised decentralisation, reduced state control, and broader participation in decision-making. In the post-Cold War period, beginning in the 1990s, health system reforms in post-conflict and fragile settings were largely driven by attempts to replicate health systems from Western countries. These reforms often overlooked the recipient countries’ existing achievements and rarely resulted in sustainable health systems and programmes that were not dependent on foreign aid. By contrast, the Yugoslav experience demonstrated that an efficient health system can be built by strengthening previously established foundations, adapting reforms to local circumstances, and recognising that there is no single model for a sustainable health system.

Yugoslav self-management evolved through repeated reforms and institutional experiments. It was a continuous process of reform, often experimental, involving governance mechanisms introduced without clear empirical evidence of their effectiveness. The underlying rationale rested on an optimistic belief in the maturation of social consciousness: that educated, empowered, and formally autonomous citizens would govern according to principles of solidarity and the common good [41]. These assumptions were deeply rooted in Marxist theories, the legacy of the wartime resistance, interwar labour movements, and the strong post-war role of trade unions. Solidarity thus became a core ideological reference point—domestically and internationally—shaping Yugoslavia’s support for decolonisation movements and its leadership role within the Non-Aligned Movement.

After the initial post-war reconstruction phase, health reforms focused mainly on two areas: financing and institutional governance. From the early 1950s, the federal state gradually retreated from direct financing and decision-making. Budgetary funding was replaced by compulsory social insurance contributions paid primarily by workers, and over time, formal control over these funds was transferred—at least nominally—to the workers themselves. At the local level, health policy and resource allocation were debated within health parliaments and, later, self-managing communities of interest, bringing together representatives of service users and providers [12,16]. Health institutions were formally governed by workers’ councils, responsible for contracting services and managing internal affairs.

Over time, policymakers increasingly recognised that health-sector development depended on broader economic conditions, and that industrial growth was prioritised through partial marketisation. This shift disadvantaged the service sector, including health care [14,44]. Health workers were often paid less than industrial workers, resources remained limited, and post-war enthusiasm gradually eroded [15]. Professional prestige and informal authority—especially within hospitals—became compensatory mechanisms, reinforcing urban concentration and contributing to persistent rural–urban disparities. Decentralisation of education further produced a cohort of locally educated professionals facing limited employment opportunities, while labour market pressures and rationalisation fostered migration and social dissatisfaction [57].

Market-oriented reforms also facilitated the emergence of a managerial–bureaucratic elite composed of enterprise managers, bankers, and administrators [14,58]. Although workers’ councils formally governed institutions, real negotiations increasingly occurred between managers, financial institutions, and state officials. In response to rising social tensions and nationalism, the Yugoslav leadership introduced a new wave of reforms culminating in the 1974 Constitution and the 1976 Law on Associated Labour. These reforms intensified decentralisation and participatory governance through the fragmentation of large enterprises into basic organisations of associated labour and the strengthening of local communities and self-managing interest communities. The explicit aim was to weaken emerging elites and deepen direct participation [14,16].

In practice, however, these measures often weakened managerial capacity without generating corresponding responsibility among workers [32]. Expectations of solidarity between profitable and loss-making units were frequently unmet, and strikes became more common. These reforms coincided with economic stagnation, global neoliberal shifts, mounting external debt, IMF conditionality, and geopolitical instability. The oil crisis of 1979 and broader crises within socialist Europe further constrained reform capacity [32]. Yet perhaps the most prominent challenges observed in the mature phase of self-management were the weakening of healthcare institutions and the almost complete erosion of national control over the healthcare system. Potential reasons for weakened institutions might have been the lack of a sense of ownership and accountability. According to Muller, shared governance should involve employees actively participating in decision-making and problem-solving, while taking responsibility and accountability for the outcomes of their decisions. Authority should be delegated to the lowest appropriate level, enabling individuals to take ownership of both successful and unsuccessful outcomes and remain accountable for their professional and organisational responsibilities [59].

On the other hand, achieving the desired level of central control of the health system is equally difficult. Even beyond health systems, an additional layer of power and control may be challenging (e.g., the contemporary role of the United Nations, WHO, or EU bodies). As noted by Rechel et al., decentralisation aims to make services more responsive to local needs and market pressures, but it cannot resolve all the tensions involved [60]. Local, person-centred decision-making can create tensions between regional fairness, responsiveness to local preferences and the efficiency benefits of economies of scale [60].

The Yugoslav experience teaches us the same lesson as the one we learnt recently during the COVID-19 pandemic: while, as a global society, we have not yet found a solution for global solidarity, the same applies to a nation—namely, the lack of solidarity between regions and administrative units. Therefore, a fundamental shift in the way we perceive society should be a prerequisite for substantial healthcare reform. Such a shift is impossible under the neoliberal, market-oriented ideology, the same ideology that destroyed Yugoslav self-management.

Atuire et al. note that efforts to advance equity-driven global health are declining and are increasingly being replaced by a transactional model shaped by geopolitical competition, conditional partnerships and a retreat from multilateralism [61]. This approach prioritises exchange, leverage and strategic interests over shared commitments to improving health for all. In this context, the survival of global health as a meaningful ethical and political endeavour is at stake. They acknowledge that “solidarity is not only a moral aspiration but also the only viable organising principle capable of drawing and sustaining collective commitment in an actively fragmenting, yet deeply interdependent, world” [61].

Tong et al. propose six strategies to enhance solidarity: a. international agreements should be greatly strengthened, and treaties and other agreements defining shared stewardship should be actively pursued; b. public health and other professionals should understand the importance of solidarity and health professionals, in particular, as stakeholders, can play a significant role in advocating for taking action to make changes and can ally themselves with other thought leaders, such as cultural and religious leaders, to emphasise solidarity; c. we should endeavour to reduce inequities within societies because the challenges need to be addressed collectively, at local, regional, national and global levels; d. trust between people and institutions, as an index of solidarity, should be cultivated and respected, and tracked as a key social indicator, and this requires controlling the spread of disinformation and countering its effects; e. we must overcome key obstacles in achieving global solidarity—geopolitical, economic, behavioural and others; the principles of mutual understanding, mutual respect and mutual support should be upheld, and collaboration and cooperation from the interpersonal to the international scales are essential; and f. we should promote a transition towards a more integral and inclusive model of development, one grounded in solidarity and responsibility [62]. While the initial main aim of these strategies is to propose actions to increase solidarity within the public health field in the context of the Anthropocene, it is equally applicable to solidarity more broadly, which is a prerequisite for effective and sustainable self-management.

Rather than leading to the “withering away” of the state, decentralisation in Yugoslavia contributed to the consolidation of republican and local political–economic elites. Competition replaced solidarity, federal institutions were increasingly perceived as adversaries, and nationalist mobilisation intensified [42]. By the late 1980s, these dynamics had become irreversible.

Classically, four forms of decentralisation that differ in the extent of authority transferred can be identified: deconcentration and delegation retain ultimate authority at the centre, while devolution grants legally independent local bodies substantial self-governing powers; dispersal involves placing central administrators locally without transferring significant authority or responsibility [63,64]. In the case of Yugoslavia, however, we need to emphasise two types of decentralisation. The first type is ‘health system governance decentralisation’ (devolution) which was reflected, on the one hand, in the very high level of autonomy enjoyed by the Yugoslav republics, provinces and local communities, on the other, in the establishment of SMICs, i.e., decentralisation to the level of a citizen. The second type is governance decentralisation within a healthcare setting (BOALs in the case of Yugoslavia). Health system governance decentralisation, in the sense of transferring greater power to ‘lower’ administrative levels (sub-national and local), has been much more extensively explored in both high-income countries and low- and middle-income countries. However, empirical evidence on whether decentralisation leads to improved health system performance remains inconclusive and controversial [65].

This Yugoslav approach, with SMICs, was not unique. Particularly in Latin America, similar efforts to promote broader participation among society and local communities can be found, for example, in Brazil and Cuba [66,67,68]. However, comparable initiatives have also been documented in other regions [69]. The main challenges associated with health governance decentralisation largely remain the same as those observed in Yugoslavia. Oliveira et al. found that decentralising health policies to municipalities can improve responsiveness and information exchange, but that its effects vary depending on socioeconomic conditions, funding, and implementation [70]. While it may promote equity when resources are distributed fairly, it can also increase costs and widen disparities, particularly disadvantaging municipalities with fewer resources. Devolution can enable local governments to tailor health systems to the needs of their populations, but it also creates differences between jurisdictions, effectively producing ‘mini’ health systems with varying levels of service provision and access [71]. This fragmentation can exacerbate existing inequalities, particularly where jurisdictions have unequal resources, making need-based resource allocation essential for maintaining equity [71].

The other form of decentralisation—within institutions—is much less explored. Even when the decentralisation of institutional governance is considered, it mainly concerns increasing healthcare institutions’ autonomy and providing them with greater direct managerial control [60]. Similarly, in the WHO’s efforts to improve participatory governance, such participation seems to remain limited to public forums and hearings, consultative meetings and discussions, and fixed seats in administrative bodies [5]. There appear to be no efforts to include healthcare workers and other staff within healthcare institutions in decision-making processes beyond purely consultative mechanisms, despite the United Nations General Assembly’s Political Declaration of the High-Level Meeting on Universal Health Coverage recognising the role of health and care workers in participatory and inclusive approaches to health governance [72]. Participatory approaches are mentioned in point 104 of the Declaration’s 109 points. This makes the ‘Yugoslav experiment’ perhaps unique in having involved each employee in decision-making within their institution.

However, this approach was echoed in Muller’s model of participative management in a healthcare organisation [59], in which participatory management is seen as a process involving collaborative decision-making and problem-solving, shared ownership and accountability among all stakeholders in the healthcare system, organisational transformation and empowerment, and effective communication within the healthcare organisation. Muller makes a point that is fully applicable to the ‘Yugoslav experiment’: “The implementation of participative management in a health care organisation poses a tremendous challenge for the health service managers. Change is never effected without difficulties, but the potential for personal, professional and organisational growth is unlimited and very worthwhile” [59].

While early literature largely celebrated Yugoslav reforms, international scholarship grew increasingly critical from the late 1970s onwards. Post-dissolution analyses often shifted towards largely unqualified condemnation. Notably, despite decades of subsequent reform, the institutional foundations of Yugoslav health systems remain largely intact across successor states, and few comparable institutional alternatives have subsequently developed.

The experience of Yugoslavia contributes to the ongoing debates on social participation for health by providing unique evidence of comprehensive social participation at the national (macro), republican and provincial (meso), and local (micro) levels over a period of nearly four decades. It may inform initiatives, including those led by the WHO [5,6], aimed at promoting social participation for health as a prerequisite for sustainable health systems. However, it also offers valuable lessons on the limitations of social participation.

Based on the Yugoslav experience, we may summarise the key lessons for the internationalisation of this unique experience in participatory governance for health:

- Decentralisation within the health system, from the central to the sub-national and local levels: This approach has already been well conceptualised, but ambiguities remain due to tensions between the need for greater participation and the need for greater efficiency [73]. Its success depends on local specificities and adequate support during the transition of power and decision-making authority. A lack of support, including financial support and capacity-building, may lead to failure, which can then be used as an argument against decentralisation and in favour of subsequent re-centralisation [74].
- Decentralisation of institutional governance, giving greater autonomy to health institutions in decision-making: The same limitations as those described for system-level decentralisation apply here [60].
- Decentralisation of governance within a health institution: The aim is to ensure that each employee, including health workers and other staff, participates in decision-making. The Yugoslav experience of BOALs may be further explored as a potential model, together with Muller’s model, which provides practical steps for establishing participatory management in health [59].
- Society and community involvement in health governance: This involvement includes both overall participation in health-system management at the local and other levels (drawing on the Yugoslav experience with SMICs and similar examples from Brazil and other countries), as well as involvement in the management of healthcare institutions, both directly (through participation in decision-making, i.e., within BOAL-equivalent managing bodies) and indirectly (through SMICs-equivalents). It should be noted that this approach would require a strategic shift away from the currently prevalent practice in which “community participation” is understood mainly as the participation of civil society organisations and representatives of the most vulnerable groups and/or patients.

This study has several limitations. The systematic literature review relied on specific search terms, which limited the analysis to studies that included these terms in titles or abstracts; relevant publications may have been missed. Grey literature was not included, which could have provided additional insights. Many studies were published several decades ago, and some were inaccessible due to availability or access restrictions. Finally, this analysis focused on selected aspects of health reform—specifically post-war recovery and self-management—while other aspects were beyond the scope of this study.

## 5. Conclusions

The Yugoslav experience with social participation for health represents one of the few attempts to reform health care through detatisation, decentralisation, depoliticisation, and democratisation, underpinned by participatory governance and solidarity. Despite structural limitations, the literature documents substantial improvements in population health, particularly during the early phases of reform. However, throughout its existence, the system remained constrained by competing economic priorities rooted in market-oriented development. The unresolved tension between these logics is widely regarded as contributing to the emergence of managerial elites and the gradual erosion of solidarity.

Several analytical lessons can be drawn from this experience. Mechanisms to limit elite capture must be built into reforms from the outset without undermining effective governance. Decentralisation must involve the genuine transfer of decision-making authority, rather than merely redistributing centres of power. Participatory governance should be paired with accountability. Finally, reforms that do not explicitly prioritise solidarity risk reproducing inequitable outcomes. The Yugoslav case therefore remains a grounded reference point for contemporary debates on sustainable social participation for health.

## Figures and Tables

**Table 1:** Phases of Health Reform in Socialist Yugoslavia.

Reform Phase	Years	Corresponding Mencinger Phase [19,20]	Key Features	Financing & Governance	Critical Junctures/Notes
Centralised-Administrative Management	1945–1952	Administrative socialism (1945–1952)	State-dominated health system; top-down planning; hospitals and clinics rebuilt postwar; early preventive care	State budget financing; workers employed by state	Tito–Stalin split (1948)—set stage for independent socialism; institutional path dependence established
Early Self-Management	1953–1960	Administrative market socialism (1953–1962)	Introduction of workers’ councils in health facilities; local autonomy begins; primary health centres established	Hybrid: central guidance + local authority; social insurance coverage expands	Expansion of local influence, but federal oversight remains strong
Formalised Self-Management	1961–1973	Market socialism (1963–1973)	Full implementation of self-management; communal insurance associations; multidisciplinary primary care teams; increased enterprise autonomy	Financing via insurance funds; state budgets gradually reduced	Standardisation of worker participation; tensions between efficiency, equity, and professional authority
Constitutional Reforms/SMICs & BOALs Era	1974–1988	Contractual socialism (1974–1988)	BOALs and SMICs introduced; complex institutional proliferation; decentralisation peaks	Local insurance bodies administer funds; participatory councils formalised	Bureaucratic fragmentation; economic stagnation; growing inequalities; nationalist tensions rise
Collapse	1989–1991	N/A	Disintegration of federal governance; health institutions increasingly unstable; coordination breaks down	Fragmented financing; SMICs and BOALs largely non-functional	Political and economic collapse; precursor to post-socialist transformations

Note: BOALs: Basic Organisations of Associated Labour; SMICs: Health Self-Management Interest Communities.

## Data Availability

No new data were created or analysed in this study. Data sharing is not applicable to this article.

## Appendix A

List of publications included in systematic review analysis

1. Albrecht, N. Peasant internationalism: Understanding social medicine beyond socialism. *Eur. J. Hist. Med. Health* **2025**, *82*(1), 13–42. https://doi.org/10.1163/26667711-bja10053.
2. Antić, A. The pedagogy of workers’ self-management: Terror, therapy, and reform communism in Yugoslavia after the Tito–Stalin split. *J. Soc. Hist.* **2016**, *50*(1), 179–203. https://doi.org/10.1093/jsh/shw013.
3. Bartlett, W.; Božikov, J.; Rechel, B. Health reforms in South East Europe: An introduction. In *Health Reforms in South East Europe*; Bartlett, W.; Božikov, J.; Rechel, B., Eds.; Palgrave Macmillan: London, UK, 2012; pp. 3–28. https://doi.org/10.1057/9781137264770_1
4. Beaven, D.W. Diabetes services in Yugoslavia. *Br. Med. J.* **1983**, *287*(6396), 894–895. https://doi.org/10.1136/bmj.287.6396.894.
5. Bogdan, B. Cold War entanglements and abortion technology: Writing Yugoslavia into the global history of vacuum aspiration, 1964–1974. *Aust. J. Polit. Hist.* **2018**, *64*(3), 407–421. https://doi.org/10.1111/ajph.12486.
6. Bogdan, B. Vračare: Village wise women, reproductive health, and Yugoslavia’s early socialist modernisation project. *J. Aging Stud.* **2023**, *64*, 101084. https://doi.org/10.1016/j.jaging.2022.101084.
7. Brujić, M. In Torlak we (would) trust: Domestic vaccine production in contemporary Serbia. *Med. Humanit.* **2022**, *48*(3), e11. https://doi.org/10.1136/medhum-2021-012212.
8. De Miguel, J.M. Policies and politics of the health reforms in southern European countries: A sociological critique. *Soc. Sci. Med.* **1977**, *11*(6–7), 379–393. https://doi.org/10.1016/0037-7856(77)90101-9.
9. Drobnič, S.; Son, K. The welfare state in Yugoslavia and its successor states: The case of family policy. In *The Generosity of Social Policies in Socialist and Post-Socialist States: Global Dynamics of Social Policy*; Heinrich, A.; Kaminska, M.E.; Pleines, H.; ten Brink, T., Eds.; Springer: Cham, Switzerland, 2025; pp. 93–118. https://doi.org/10.1007/978-3-031-87733-9_5.
10. Durlević, Z. Razvoj zdravstvene delatnosti u drugoj Jugoslaviji od 1944. do 1991. godine. *Naučni Časopis Urgentne Medicine HALO 194* **2013**, 19(2), 78–89. (In Serbian).
11. Estrin, S. Yugoslavia: The case of self-managing market socialism. *J. Econ. Perspect.* **1991**, *5*(4), 187–194. https://doi.org/10.1257/jep.5.4.187
12. Grielen, S.J.; Boerma, W.G.W.; Groenewegen, P.P. Unity or diversity? Task profiles of general practitioners in Central and Eastern Europe. *Eur. J. Public Health* **2000**, *10*(4), 249–254. https://doi.org/10.1093/eurpub/10.4.249.
13. Hill, E.; Jankovic, S. Revitalizing health promotion: A case study from Serbia. *Promot. Educ.* **2006**, *13*(4), 243–247. https://doi.org/10.1177/175797590601300403.
14. Himmelstein, D.U.; Lang, S.; Woolhandler, S. The Yugoslav health system: Public ownership and local control. *J. Public Health Policy* **1984**, *5*(3), 423–431.
15. Kessler, H.H. Medicine in Yugoslavia. *JAMA* **1951**, *147*(16), 1564–1567. https://doi.org/10.1001/jama.1951.73670330011013.
16. Kunitz, S.J. Health care and workers’ self-management in Yugoslavia. *Int. J. Health Serv.* **1979**, *9*(3), 521–537. https://doi.org/10.2190/9240-YUX2-VX2H-RU0L.
17. Kunitz, S.J. The recruitment, training, and distribution of physicians in Yugoslavia. *Int. J. Health Serv.* **1980**, *10(4)*, 587–609. https://doi.org/10.2190/0Q25-HLXU-KGRE-2C5N.
18. Kunitz, S.J. What Yugoslavia means: Progress, nationalism, and health. *Health Transit. Rev.* **1996**, *6*(Suppl.), 253–272.
19. Kunitz, S.J. The making and breaking of Yugoslavia and its impact on health. *Am. J. Public Health* **2004**, *94*(11), 1894–1904. https://doi.org/10.2105/AJPH.94.11.1894.
20. Liotta, P.H. Paradigm lost: Yugoslav self-management and the economics of disaster. *Balkanologie* **2001**, *5*(1–2). https://doi.org/10.4000/balkanologie.681.
21. Mastilica, M. Health and social inequities in Yugoslavia. *Soc. Sci. Med.* **1990**, *31*(3), 405–412. https://doi.org/10.1016/0277-9536(90)90287-3.
22. Milanović, V. Humanism and medicine. *Soc. Sci. Med.* **1987**, *25*(1), 35–41. https://doi.org/10.1016/0277-9536(87)90201-2.
23. Milenkovitch, D.D. The case of Yugoslavia. *Am. Econ. Rev.* **1977**, *67*(1), 55–60.
24. Mowlem, R. Medicine in Yugoslavia. *Br. Med. J.* **1950**, *1*(4661), 1072–1073.
25. Novosel, M.; Kohn, R. Physician and society: A study of people’s perception of the social role of physicians in a region of Yugoslavia. *Soc. Sci. Med.* **1979**, *13*, 73–80. https://doi.org/10.1016/0271-7123(79)90010-5.
26. Parmelee, D.E. Whither the state in Yugoslav health care? *Soc. Sci. Med.* **1985**, *21*(7), 719–728. https://doi.org/10.1016/0277-9536(85)90116-9.
27. Parmelee, D.E.; Henderson, G.; Cohen, M.S. Medicine under socialism: Some observations on Yugoslavia and China. *Soc. Sci. Med.* **1982**, *16*(15), 1389–1396. https://doi.org/10.1016/0277-9536(82)90133-2.
28. Popić, T.; Perišić, N. Former Yugoslavia. In *Health Politics in Europe: A Handbook*; Immergut, E.M.; Anderson, K.M.; Devitt, C.; Popić, T., Eds.; Oxford University Press: Oxford, UK, 2021; pp. 901–907. https://doi.org/10.1093/oso/9780198860525.003.0040.
29. Popić, T. Slovenia. In *Health Reforms in Post-Communist Eastern Europe*; Popić, T., Ed.; Springer: Cham, Switzerland, 2023; pp. 59–104. https://doi.org/10.1007/978-3-031-15497-3_3.
30. Romano, J. Pukovnik pregled lekara, farmaceuta, studenata medicine i farmacije učesnika NOR-a i poginulih u ratu, učešće i doprinos bolničarki u NOV i POJ 1941–1945. In *Sanitetska služba u Narodnooslobodilačkom ratu 1941–1945*, Vol. IV; Vojnoizdavački i novinski centar: Beograd, Serbia, 1989. (In Serbian).
31. Sarić, M.; Rodwin, V.G. The once and future health system in the former Yugoslavia: Myths and realities. *J. Public Health Policy* **1993**, *14*(2), 220–237. https://doi.org/10.2307/3342966.
32. Savelli, M. Peace and happiness await us: Psychotherapy in Yugoslavia, 1945–1985. *Hist. Hum. Sci.* **2018**, *31*(4), 38–57. https://doi.org/10.1177/0952695118773951.
33. Savelli, M. Socialism, society, and the struggle against mental illness: Preventative psychiatry in post-war Yugoslavia. In *Preventing Mental Illness: Mental Health in Historical Perspective*; Kritsotaki, D.; Long, V.; Smith, M., Eds.; Springer: Cham, Switzerland, 2019; pp. 131–150. https://doi.org/10.1007/978-3-319-98699-9_6.
34. Savelli, M. The afterlives of mental hygiene: Psychiatrizing society in socialist Yugoslavia. *Soc. Hist. Med.* **2024**, *37*(2), 364–386. https://doi.org/10.1093/shm/hkac067.
35. Simić, I. The curious case of Aleksandar Milivojević: The Donja Toponica Hospital and mental health in socialist Yugoslavia. *Soc. Hist. Med.* **2020**, *33*(4), 1350–1362. https://doi.org/10.1093/shm/hkz033.
36. Simić, S.; Santrić Milićević, M.; Matejić, B.; Marinković, J.; Adams, O. Do we have primary health care reform? The story of the Republic of Serbia. *Health Policy* **2010**, *96*, 160–169. https://doi.org/10.1016/j.healthpol.2010.01.015.
37. Švab, I. Primary health care reform in Slovenia: First results. *Soc. Sci. Med.* **1995**, *41*(1), 141–144. https://doi.org/10.1016/0277-9536(94)00323-L.
38. Spasenoska, D. Dynamics of life expectancy change during the transition to democracy in the ex-Yugoslav countries. *Espace Popul. Soc.* **2022**, *2-3*. https://doi.org/10.4000/eps.12945.
39. Vukmanović, C. Decentralized socialism: Medical care in Yugoslavia. *Int. J. Health Serv.* **1972**, *2*(1), 35–44. https://doi.org/10.2190/Q611-5JHT-6691-N1CU.
40. Zrinščak, S. Zdravstvena politika Hrvatske: U vrtlogu reformi i suvremenih društvenih izazova. *Rev. Soc. Polit.* **2007**, *14*(2), 193–220. (In Croatian). https://doi.org/10.3935/rsp.v14i2.697.

## Appendix B

List of publications excluded from the analysis (not retrieved or do not meet the inclusion criteria)

1. Belson, D.L. Self-managed health care in Yugoslavia. *Health Care Manage Rev.* **1977**, *2*(1), 71–75. https://doi.org/10.1097/00004010-197700210-00012.
2. Bjegovic-Mikanovic, V.; Vukovic, D.; Terzic-Supic, Z.; Santric-Milicevic, M.S.; Laaser, U. Strategic orientation of public health in transition: An overview of South Eastern Europe. *J. Public Health Policy* **2007**, *28*(1), 94–101. https://doi.org/10.1057/palgrave.jphp.3200121.
3. Blaskovich, J.N. Organizational structure of American medical practice: Health care crisis and reform. *Croat. Med. J.* **1994**, *35*(3), 137–142.
4. Boerma, W.G.W. Profiles of General Practice in Europe: An International Study of Variation in the Tasks of General Practitioners. Doctoral Thesis, Maastricht University, Maastricht, The Netherlands, 17 September 2003. https://doi.org/10.26481/dis.20030917wb
5. Bojanin, S. A mental hygienic approach to adolescent age (Serbocroatian). *Anali Zavoda za mentalno zdravlje* **1973**, *5*(4), 105–116.
6. Bundy, A.M. *There Was a Man of UNRRA: Internationalism, Humanitarianism, and the Early Cold War in Europe, 1943–1947*. Doctoral Thesis, The Ohio State University, Columbus, OH, USA, 2017. Available online: https://etd.ohiolink.edu/acprod/odb_etd/ws/send_file/send?accession=osu1492608585636304&disposition=inline (accessed on 20 January 2026).
7. Cesen, M.; Drnovsek, VM. The process of health legislation reform in the Republic of Slovenia. *Eur. J. Health Law* **2000**, *7*(1), 73–84. https://doi.org/10.1163/15718090020523061.
8. Clark, C. Government spending, policy outputs, and policy outcomes: Health policy in Yugoslavia. *Environ. Plann.* C *Gov. Policy* **1990**, *8*(2), 123–138. https://doi.org/10.1068/c080123
9. Čatić, T.; Tabakovic, V.; Vuk, S.M.; Bejtovic, H.; Kopanja, D.; Samardzic, D.; Skrbo, A.; Mačić, I. Historical developments of Bosnia and Herzegovina pharmaceutical industry—The past and the future perspectives of domestic manufacturing. *Mater. Sociomed.* **2022**, *34*(3), 228–235. https://doi.org/10.5455/msm.2022.34.228-235.
10. Čavoški, J. And after Tito… no one. Yugoslavia and the crisis of the Non-Aligned Movement during the early 1980s. *Istorija 20. Veka* **2024**, *42*(1), 233–256. https://doi.org/10.29362/ist20veka.2024.1.cav.233-256.
11. Dugac, Ž. Croatian Medical Association, Andrija Štampar and public health politics in the Kingdom of Serbs, Croats and Slovenians (Kingdom of Yugoslavia). *Liječnički Vjesnik* **2005**, *127*(5-6), 151–157.
12. Dugac, Ž. Building an original and internationally standardized public health system in the Kingdom of Yugoslavia. *Acta Historica Medicinae, Stomatologiae, Pharmaciae, Medicinae Veterinariae* **2024**, *43*(1), 51–64. https://doi.org/10.5937/acthist43-52883.
13. Dugački, V.; Regan, K. Povijest zdravstvene skrbi i razvoja zdravstvenih ustanova na hrvatskom prostoru. *Studia Lexicographica* **2019**, *13*(25), 35–74. https://doi.org/10.33604/sl.13.25.2.
14. Echols, J.M. Racial and ethnic inequality: The comparative impact of socialism. *Comp. Polit. Stud.* **1981**, *13*(4), 403–444. https://doi.org/10.1177/001041408101300403.
15. Ensor, T.; Thompson, R. Health insurance as a catalyst to change in former communist countries? *Health Policy* **1998**, *43(3)*, 203–218. https://doi.org/10.1016/s0168-8510(97)00098-5
16. Ferhadbegović, S. Past and Present Health Crises: How Yugoslavia Managed the Smallpox Epidemic of 1972. *Cultures of History Forum* **2020**. Available online: https://www.cultures-of-history.uni-jena.de/focus/kleio-in-pandemia/past-and-present-health-crises-how-yugoslavia-managed-the-smallpox-epidemic-of-1972/ (accessed on 19 January 2026)
17. Gajić-Stevanović, M.; Stevanović, I. Overview of changes in the health sector and its financing in the Republic of Serbia in the period 2004–2020. *Serbian Dental Journal* **2023**, *70*(2), 93–101. https://doi.org/10.2298/SGS2302095G.
18. Geisthövel, A. Exploring state socialist governmentality: Eastern European examples from medicine and health care. *Hist. Human Sci.* **2026**, *39*(2), 3–20. https://doi.org/10.1177/09526951251385887.
19. Healy, J.; McKee, M. Implementing hospital reform in central and eastern Europe. *Health Policy* **2002**, *61(1)*, 1–19. https://doi.org/10.1016/s0168-8510(01)00213-5
20. Ibrahim, KM.; Yassin, K.M.; Khan, S.; Laaser, U. Decline in the child health indicators: Assessing the impact of war in five South-Eastern European countries. *Period. Biol.* **2003**, *105*(1), 37–43.
21. Iacob, B.C. Malariology and decolonization: Eastern European experts from the League of Nations to the World Health Organization. *J. Glob. Hist.* **2022**, *17*(2), 233–253. https://doi.org/10.1017/S1740022822000067.
22. Jakovljevic, M.M.; Arsenijevic, J.; Pavlova, M.; Verhaeghe, N.; Laaser, U.; Groot, W. Within the triangle of healthcare legacies: Comparing the performance of South-Eastern European health systems. *J. Med. Econ.* **2017**, *20*(5), 483–492. https://doi.org/10.1080/13696998.2016.1277228.
23. Jakovljevic, M. Resource allocation strategies in Southeastern European health policy. *Eur. J.* Health *Econ.* **2013**, *14*(2), 153–159. https://doi.org/10.1007/s10198-012-0439-y.
24. Karge, H. Transnational knowledge into Yugoslav practices? The legacy of the Second World War on social welfare policy in Yugoslavia. *Comparativ* **2010**, *20*(5), 75–86.
25. Klančar, D.; Švab, I. Primary care principles and community health centers in the countries of former Yugoslavia. *Health Policy* **2014**, *118*(2), 166–172. https://doi.org/10.1016/j.healthpol.2014.08.014.
26. Klančar, D. Primary Health Care Organisation in the Countries in the Area of Yugoslavia.Doctoral dissertation, University of Ljubljana, Ljubljana, Slovenia, 2021. (In Slovenian)
27. Komadina, D. Integration of consumers and providers of health services. *World Hosp.* **1976**, *12*(1), 40–43.
28. Kostić, M.; Vuković, D. Regression of hard-won advances in socialized medicine: The emergence of the private sector in health care in Serbia. *Health Hum. Rights.* **2025**, *27*(2), 229–242.
29. Kronja, T. Ethical problems of health and health workers. *Liječnički Vjesnik* **1969**, *91*(6), 581–588.
30. Kutzin, J.; Jakab, M.; Cashin, C. Lessons from health financing reform in central and eastern Europe and the former Soviet Union. *Health Econ. Policy Law* **2010**, *5*, 135–147. https://doi.org/10.1017/S1744133110000010.
31. Mardesić D, Gjurić G, Jezerinac Z. Sistematsko traganje za nasljednim metaboličkim bolestima u svijetu i u nas [Screening for inborn errors of metabolism in the newborn population abroad and in our country (author’s transl)]. Lijec Vjesn. **1979**, *101*(12):746–750.
32. McCarthy, M. Serbia rebuilds and reforms its health-care system. *The Lancet* **2007**, *369*(9559), 360. https://doi.org/10.1016/S0140-6736(07)60173-1.
33. Miller, N. Return engagement: Intellectuals and nationalism in Tito’s Yugoslavia. In *State Collapse in South-Eastern Europe: New Perspectives on Yugoslavia’s Disintegration*; Cohen, L.J., Dragović-Soso, J., Eds.; Purdue University Press: West Lafayette, IN, USA, 2008; pp. 179–200.
34. Miller, R.F. Social, economic and political dilemmas of reform in Yugoslavia. *Politics* **1989**, 24(1), 92–107. https://doi.org/10.1080/00323268908402081.
35. Mladenović, D.; Simić, S. All Health Care System Reforms in Former and Present Yugoslavia. In *Osnove za reformu sistema zdravstvene zaštite u Republici Srbiji*; Centar za proučavanje alternativa: Beograd, Serbia, 2001; pp. 33–45.
36. Nikolic, B. Historical and modern development of healthcare in Čazma. *Radovi Zavoda za Znanstvenoistraživački i Umjetnički Rad u Bjelovaru* **2019**, *13*, 107–114. https://doi.org/10.21857/MNLQGC036Y.
37. Nolimal, D. Some reflections on alcohol public policy in Yugoslavia. *Socijalna Psihijatrija* **1989**, *17*(1), 67–82.
38. Orešković, S. Health system reorganization in Croatia in the light of major reform tendencies in OECD countries. *Croat. Med. J.* **1995**, *36*(1), 47–54.
39. Ovseiko, P.V. The Politics of Health Care Reform in Central and Eastern Europe: The Case of the Czech Republic. Doctoral Thesis, University of Oxford, Oxford, UK, 2009. https://doi.org/10.5287/ora-nb98kn0p4.
40. Parmelee, D.E. Yugoslavia: Health Care under Self-Managing Socialism. In Success and Crisis in National Health Systems: A Comparative Approach; Field, M.G., Ed.; Routledge: New York, NY, USA, London, UK, 1989; pp. 165–191.
41. Pavlínek, P.; Pickles, J. Part II: Nature, risk and the legacies of state socialism. In *Environmental Transitions: Transformation and Ecological Defense in Central and Eastern Europe*; Routledge: London/New York, 2000; pp. 37–154.
42. Pašić, N. The political articulation and aggregation of plural interests in self-management systems. Wilson Center Fellowship Report, 1986.
43. Skubic Ermenc, K.; Bembič, B. Slovenia: Segmentation and Sectoral Disparities. In Skill Formation in Central and Eastern Europe: A Search for Patterns and Directions of Development; Tūtlys, V., Markowitsch, J., Pavlin, S., Winterton, J., Eds.; Peter Lang: Berlin, Germany, 2022; pp. 243–264.
44. Perlmutter, F.D. Citizen participation in Yugoslavia. *Social Work* **1974**, *19*(2), 226–232. https://doi.org/10.1093/sw/19.2.226.
45. Popovic, B.; Skribic, M.; Kohn, R. Yugoslavia’s autonomous health institutions. Their role within the constitutional and sociopolitical framework in the Republic of Croatia. *International Journal of Health Services* **1973**, *3*(2), 213–221. https://doi.org/10.2190/BPBP-4KED-TW3H-F7P4.
46. Prica, V.P. The response of Yugoslav architects on the health challenges and crisis 1918–1941. *Istorija 20. Veka* **2023**, *41*(1), 45–66. https://doi.org/10.29362/ist20veka.2023.1.put.45-66.
47. Radovanović, M. Desirable ways of reforming the health care system in Yugoslavia. *Med. Pregl.* **1994**, *47*(3-4), 77–78.
48. Rajić, Z.; Gorski, B. Zagreb hospitals at a turning point: A comprehensive analysis and reorganization proposals with a key role for the University Hospital in Novi Zagreb *Croat.* Med. *J.* **1994**, *35*(4), 203–213.
49. Rechel, B.; McKee, M. Health reform in central and eastern Europe and the former Soviet Union. *The Lancet* **2009**, *374*, 1186–1195. https://doi.org/10.1016/S0140-6736(09)61334-9
50. Rusinow, D. Understanding the Yugoslav reforms. *The World Today* **1967**, *23*(2), 71–79.
51. Souliotis, K.; Economou, C.; Tountas, Y. Health care reforms in the ex-socialist South-Eastern European countries. *J. Soc. Soc. Policy* **2005**, *4*(1), 63–76.
52. Spasić, I. Yugoslavia as a place for living a normal life: Memories of ordinary people in Serbia. *Sociologija* **2012**, *54*(4), 577–594. https://doi.org/10.2298/SOC1204577S.
53. Spaskovska, L. Constructing the “City of International Solidarity”: Non-Aligned internationalism, the United Nations and visions of development, modernism and solidarity, 1955–1975. *Journal of World History* **2020**, *31(1)*, 137–164.
54. Stanimirović, N. (2021). Self-managing the brotherhood and unity: Understanding Tito’s approach to nationalism.Master’s thesis, Budapest, Hungary, Central European University.
55. Stojanović, R. The sources of contradiction in self-managing socialism. *Zbornik Radova* **1989**, *23*(1–3), 125–131.
56. Tavanxhi, N.; Burazeri, G.; Laaser, U. Southeastern Europe, health systems of. In *International Encyclopedia of Public Health*; Heggenhougen, H.K., Ed.; Academic Press: Oxford, UK, 2008; pp. 137–147. https://doi.org/10.1016/B978-012373960-5.00308-7.
57. Toth, M. Group practice in Yugoslavia. *International Dental Journal* **1986**, *36*(2), 102–105.
58. Uvalić, M. The rise and fall of market socialism in Yugoslavia. 2019.
59. Vlahović, Z.; Radojković, D. Healthcare in Serbia in transition period. *EPMA J.* **2010**, *1*(4), 601–606. https://doi.org/10.1007/s13167-010-0055-9.
60. Živković, M.; Bjegović, V.; Simić, S.; Marinković, J. Laboratory testing in primary health care in Belgrade. International Journal for Quality in Health Care 1994, 6(1), 97–102. https://doi.org/10.1093/intqhc/6.1.97.

## Appendix C

**Table A1:** Characteristics of included publications (*n* = 40).

Author(s), Year	Source Type	Nature of Contribution	Temporal Context	Health-System Domain	Evidentiary Role *
Albrecht, 2025 [27]	Article	Historical	1920s–1950s: Pre-war & Centralised administrative management	Social medicine	Primary
Antić, 2016 [22]	Article	Historical	1950s: Early self-management	Psychiatry	Contextual
Bartlett et al., 2012 [34]	Chapter	Historical	Multi-period (1960s–1980s)	Health system reform	Primary
Beaven, 1983 [47]	Article	Report	1974–1988: Constitutional Reforms/SMIC & BOAL Era	Diabetes services	Contextual
Bogdan, 2018 [24]	Article	Historical	1964–1974: Formalised self-management	Reproductive health	Contextual
Bogdan, 2023 [30]	Article	Historical	Late 1940s–1950s: Centralised administrative management	Reproductive health	Contextual
Brujić, 2022 [45]	Article	Empirical	1974–1988 → post-1991: Constitutional Reforms/SMICs & BOAL Era → Post-socialist transition	Vaccines	Contextual
De Miguel, 1977 [13]	Article	Theoretical	Multi-period (1947–1970)	Health policy and reform	Primary
Drobnič & Son, 2025 [17]	Chapter	Historical/Analytical	Multi-period (Interwar-post-socialist period)	Family policy	Contextual
Durlević, 2013 [33]	Article	Historical	Multi-period (1944–1991)	Health services development	Primary
Estrin, 1991 [32]	Article	Theoretical	Multi-period (1952–1980)	Market socialism	Primary
Grielen et al., 2000 [37]	Article	Empirical	1990s: Post-socialist transition (1951–post-socialist period)	Primary care	Contextual
Hill & Jankovic, 2006 [39]	Article	Analytical	Multi-period (1951–post-socialist period)	Health education and promotion	Contextual
Himmelstein et al., 1984 [40]	Article	Historical	Multi-period (Interwar period–1984)	Health system	Primary
Kessler, 1951 [26]	Article	Report	1945–1950: Centralised administrative management	System organization	Primary
Kunitz, 1979 [15]	Article	Historical/Analytical	Multi-period (Post-war period–1976)	Health care self-management	Primary
Kunitz, 1980 [46]	Article	Historical/Empirical	Multi-period (Interwar period–1970s)	Health workforce	Primary
Kunitz, 1996 [42]	Article	Empirical	Multi-period (1940s–1992)	Population health	Primary
Kunitz, 2004 [57]	Article	Empirical	Multi-period (1940s–1999)	Health outcomes	Contextual
Liotta, 2001 [51]	Article	Historical/Analytical	Multi-period (1945–1990s)	Economic governance	Primary
Mastilica, 1990 [52]	Article	Empirical	Multiperiod (1950s–1980s)	Equity	Contextual
Milanović, 1987 [58]	Article	Viewpoint	1974–1987: Constitutional Reforms/SMIC & BOAL Era	Health system/governance	Primary
Milenkovitch, 1977 [14]	Article	Historical/Analytical	1968–1970: Formalised self-management; Constitutional Reforms/SMIC & BOAL Era	Political economy	Primary
Mowlem, 1950 [29]	Article	Report	1945–1950: Centralised administrative management	Medical services	Primary
Novosel & Kohn, 1979 [50]	Article	Empirical	1961–1973: Formalised self-management	Medical profession	Contextual
Parmelee et al., 1982 [38]	Article	Comparative/Analytical	1977–1982: Constitutional Reforms/SMIC & BOAL Era	Health systems	Primary
Parmelee, 1985 [16]	Article	Historical/Analytical	Multi-period (1945–1982)	Health systems	Primary
Popić & Perišić, 2021 [35]	Chapter	Historical	Multi-period (Late 19th century–1980s)	Health systems	Primary
Popić, 2023 [25]	Chapter	Historical	Multi-period (1945–post-socialist period)	System governance	Primary
Romano, 1989 [21]	Chapter	Historical	1941–1945 Health service in WWII	Healthcare workforce	Contextual
Sarić & Rodwin, 1993 [44]	Article	Historical	1974–1988: Constitutional Reforms/SMIC & BOAL Era	Health systems	Primary
Savelli, 2018 [23]	Article	Historical	Multi-period (1945–1985)	Mental health	Contextual
Savelli, 2019 [31]	Chapter	Historical	Multi-period (1945–1980s)	Mental health	Contextual
Savelli, 2024 [41]	Article	Historical	Multi-period (Interwar period–1980s)	Mental health	Contextual
Švab, 1995 [36]	Article	Historical/Analytical	Multi-period (Interwar period–1980s)	Primary care	Primary
Simić, 2020 [49]	Article	Historical	1950s: Early self-management	Mental health institutions	Contextual
Simić et al., 2010 [48]	Article	Empirical	Multi-period (1930s–post-socialist period)	Primary care	Contextual
Spasenoska, 2022 [43]	Article	Empirical	1990s–2010s: Post-socialist transition	Population health	Contextual
Vukmanović, 1972 [12]	Article	Theoretical	1953–1960: Early self-management	Medical care organisation	Primary
Zrinščak, 2007 [28]	Article	Historical/Analytical	Multi-period (19th century–2006)	Health policy	Primary

* Contextual studies were eligible if they either provided in-depth analysis of specific services or specialties—including mental health, reproductive health, vaccines, or primary care—relevant to understanding reform trajectories, institutional continuity, or legacies of self-management, or provided background information on health system reform between 1945 and 1991. Note: BOAL: Basic Organisations of Associated Labour; SMIC: Health Self-Management Interest Communities.

## Appendix D

**Table A2:** PRISMA Checklist.

Section and Topic	Item #	Checklist Item	Location Where Item Is Reported
TITLE			
Title	1	Identify the report as a systematic review.	Title
ABSTRACT			
Abstract	2	See the PRISMA 2020 for Abstracts checklist.	Abstract (pp. 1–2)
INTRODUCTION			
Rationale	3	Describe the rationale for the review in the context of existing knowledge.	Introduction (pp. 2–3)
Objectives	4	Provide an explicit statement of the objective(s) or question(s) the review addresses.	Introduction (pp. 2–3)
METHODS			
Eligibility criteria	5	Specify the inclusion and exclusion criteria for the review and how studies were grouped for the syntheses.	Materials and methods (Eligibility criteria, p. 4)
Information sources	6	Specify all databases, registers, websites, organisations, reference lists and other sources searched or consulted to identify studies. Specify the date when each source was last searched or consulted.	Materials and methods (Search Strategy, p. 4)
Search strategy	7	Present the full search strategies for all databases, registers and websites, including any filters and limits used.	Materials and methods (Search Strategy, p. 4)
Selection process	8	Specify the methods used to decide whether a study met the inclusion criteria of the review, including how many reviewers screened each record and each report retrieved, whether they worked independently, and if applicable, details of automation tools used in the process.	Materials and methods (Screening and Data Extraction, p. 5)
Data collection process	9	Specify the methods used to collect data from reports, including how many reviewers collected data from each report, whether they worked independently, any processes for obtaining or confirming data from study investigators, and if applicable, details of automation tools used in the process.	Materials and methods (Screening and Data Extraction, p. 5)
Data items	10a	List and define all outcomes for which data were sought. Specify whether all results that were compatible with each outcome domain in each study were sought (e.g., for all measures, time points, analyses), and if not, the methods used to decide which results to collect.	Materials and methods (Data synthesis and analysis, p. 6)
	10b	List and define all other variables for which data were sought (e.g., participant and intervention characteristics, funding sources). Describe any assumptions made about any missing or unclear information.	Materials and methods (Data synthesis and analysis, p. 6)
Study risk of bias assessment	11	Specify the methods used to assess risk of bias in the included studies, including details of the tool(s) used, how many reviewers assessed each study and whether they worked independently, and if applicable, details of automation tools used in the process.	Materials and methods (Evidence Classification and Appraisal, p. 5)
Effect measures	12	Specify for each outcome the effect measure(s) (e.g., risk ratio, mean difference) used in the synthesis or presentation of results.	N/A
Synthesis methods	13a	Describe the processes used to decide which studies were eligible for each synthesis (e.g., tabulating the study intervention characteristics and comparing against the planned groups for each synthesis (item #5)).	Materials and Methods (Data Synthesis and Analysis, p. 6)
	13b	Describe any methods required to prepare the data for presentation or synthesis, such as handling of missing summary statistics, or data conversions.	N/A
	13c	Describe any methods used to tabulate or visually display results of individual studies and syntheses.	N/A
	13d	Describe any methods used to synthesize results and provide a rationale for the choice(s). If meta-analysis was performed, describe the model(s), method(s) to identify the presence and extent of statistical heterogeneity, and software package(s) used.	Materials and Methods (Data Synthesis and Analysis, p. 6)
	13e	Describe any methods used to explore possible causes of heterogeneity among study results (e.g., subgroup analysis, meta-regression).	N/A
	13f	Describe any sensitivity analyses conducted to assess robustness of the synthesized results.	N/A
Reporting bias assessment	14	Describe any methods used to assess risk of bias due to missing results in a synthesis (arising from reporting biases).	N/A
Certainty assessment	15	Describe any methods used to assess certainty (or confidence) in the body of evidence for an outcome.	N/A
RESULTS			
Study selection	16a	Describe the results of the search and selection process, from the number of records identified in the search to the number of studies included in the review, ideally using a flow diagram.	Results (p. 6)
	16b	Cite studies that might appear to meet the inclusion criteria, but which were excluded, and explain why they were excluded.	Figure 1, Appendix B (p. 21)
Study characteristics	17	Cite each included study and present its characteristics.	Appendix A (p. 19), Appendix C (p. 25)
Risk of bias in studies	18	Present assessments of risk of bias for each included study.	N/A
Results of individual studies	19	For all outcomes, present, for each study: (a) summary statistics for each group (where appropriate) and (b) an effect estimate and its precision (e.g., confidence/credible interval), ideally using structured tables or plots.	N/A
Results of syntheses	20a	For each synthesis, briefly summarise the characteristics and risk of bias among contributing studies.	N/A
	20b	Present results of all statistical syntheses conducted. If meta-analysis was done, present for each the summary estimate and its precision (e.g., confidence/credible interval) and measures of statistical heterogeneity. If comparing groups, describe the direction of the effect.	N/A
	20c	Present results of all investigations of possible causes of heterogeneity among study results.	N/A
	20d	Present results of all sensitivity analyses conducted to assess the robustness of the synthesized results.	N/A
Reporting biases	21	Present assessments of risk of bias due to missing results (arising from reporting biases) for each synthesis assessed.	N/A
Certainty of evidence	22	Present assessments of certainty (or confidence) in the body of evidence for each outcome assessed.	N/A
DISCUSSION			
Discussion	23a	Provide a general interpretation of the results in the context of other evidence.	Discussion (pp. 12–17)
	23b	Discuss any limitations of the evidence included in the review.	Discussion (pp. 12–17)
	23c	Discuss any limitations of the review processes used.	Discussion (pp. 12–17)
	23d	Discuss implications of the results for practice, policy, and future research.	Conclusion (p. 17)
OTHER INFORMATION			
Registration and protocol	24a	Provide registration information for the review, including register name and registration number, or state that the review was not registered.	N/A
	24b	Indicate where the review protocol can be accessed, or state that a protocol was not prepared.	Materials and Methods, Data Synthesis and Analysis (p. 6)
	24c	Describe and explain any amendments to information provided at registration or in the protocol.	N/A
Support	25	Describe sources of financial or non-financial support for the review, and the role of the funders or sponsors in the review.	Funding (p. 18)
Competing interests	26	Declare any competing interests of review authors.	Declaration of Conflict of Interest (p. 18)
Availability of data, code and other materials	27	Report which of the following are publicly available and where they can be found: template data collection forms; data extracted from included studies; data used for all analyses; analytic code; any other materials used in the review.	Data Availability Statement, Supplement (p. 18)
